# Editorials

**Published:** 1912-09

**Authors:** 


### EDITORIAL

The Business Office of the *Journal-Record* is 23 West Alabama Street.
The Editorial Office is 1014-15 Atlanta National Bank Building.
Address all Business Communications to *Journal-Record of Medicine,* 23 West Alabama Street.
Make remittances payable to *The Journal-Record of Medicine.*
On matters pertaining to the Editorial and Original Communications, address Edgar G. Ballenger, M. D., Atlanta, Ga.
Reprints of Original Articles will be furnished at cost price. Requests foi the same should always be made in THE MANUSCRIPT.
We will present, postpaid, on request to each contributor of an original article, twenty (20) marked copies of *The Journal-Record of Medicine* containing such article.

### DRUG HABIT.

When cocaine was given to the profession in the early eighties, it was welcomed with acclaim because of its anaesthetic and ischaemic properties which enabled the surgeon to perform operations that had seemed impossible on account of the pain and blood, no matter how desirable they were in the way of relieving the patients. The freeing of the nasal passages in cases of hay-fever seemed a veritable God-send while the experiments were young. Other properties of the drug soon became evident and many physicians, through desiring to investigate them for themselves, found the worst effect and became habitues. Since then the drug has been known more for its habit-forming poison and the frightful disasters it has caused than for the good it has done. Surgeons have striven to get away from its use as far as possible, and for a man to prescribe it and allow the patient to administer it to himself in any form, is looked upon as poor practice. So much misery has been caused by that drug that it has been said that the profession has learned its lesson and will never take up a similar poison with the risk of forming a habit in any patient.

And yet here is another drug warning its users by its very alkaloidal character and having no such striking claims of good work as cocaine had in the beginning, which already has estabished itself among the worst of the habit-forming poisons.

Heroin Hydrochloride, a derivative of Morphine, a constituent of many cough mixtures, proprietary and otherwise, is listed in Merck's Manual with a dose of 1-12 to 1-6 of a grain to be used in cases of bronchitis and asthma as a sedative. It has no other claim; it is in no wise curative; it is simply a slovenly palliative, given only to relieve a pain that requires more careful treatment. It has no dignity of presence nor nobility of existence. It sneaks in, and what is the result?

At a meeting of the Fulton County Medical Society held recently, it was shown in a paper by Dr. J. C. King that it was the worst of the habit-forming drugs that he had to contend with in his practice. It resisted all the methods of relief applied to morphine and cocaine patients and was far more degrading upon the mental and physical conditions of its victims than all the other drugs. Not only this, but the dose required is small; many habitues being contented with the 1-12 or the 1-6 of a grain three or four times a day, as put down in the books as the therapeutic dose. In the discussion several physicians recalled cases of patients desiring refills of cough mixtures that contained heroin when there seemed to be no need of them.

We have ourselves experienced the depressing effects of the drug and have been alarmed at its action in the 1-12 grain dose.

It is high time that everyone should be alarmed at the presence of this drug in our medicaments. Apply the old rule and never prescribe it unless the effect is accurately understood and specifically desired. This, in our judgment, will put the drug off the list and prevent the formation of a most unfortunate habit among many people. Certainly the ordering of a nicely colored and pleasant tasting cough mixture containing Heroin is to be strongly reprehended and is not the practice of a thoughtful and careful physician.

